# Omphalocele with Intra Abdominal Anomalies

**Published:** 2014-01-01

**Authors:** Muataz A. Al Ani, Safira A. Ali Khan

**Affiliations:** Pediatric Surgery Center, Al Khansaa Teaching Hospital, Mosul – IRAQ

**Keywords:** Omphalocele, Multicystic kidney, Duplication cyst

## Abstract

Abdominal wall defects are associated with other intra-abdominal anomalies. We report two neonates with omphalocele associated with intra-abdominal anomalies. One neonate had multicystic kidney. Other neonate had duplication cyst of ileum which was missed during initial closure in neonatal life.

## INTRODUCTION

The birth prevalence of omphalocele is 1 in 5000 births. More than 50% of cases have other serious defects involving the alimentary tract and cardiovascular, genitourinary, musculo-skeletal, and central nervous systems. Chromosomal anomalies are seen in 30-40% cases of omphalocele [1-3]. We report two neonates with omphalocele associated with intra-abdominal pathology. 

## CASE REPORT

**Case 1:** A newborn baby presented with giant omphalocele (7x8 cm), (Fig. 1) with no other gross associated anomalies. The baby passed meconium within the first 24 hours of life. Ultrasound examination revealed abnormal right kidney consisted of multiple cysts of 10x12 cm in total. Echocardiogram was normal. At operation, facial closure of the defect was possible after nephrectomy for multicystic right kidney (Fig. 2). The histopathological examination confirmed the diagnosis of multicystic dysplastic kidney.

**Figure:**
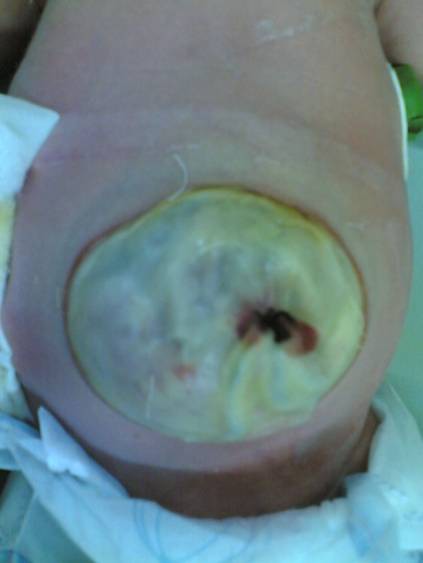
Figure 1: Omphalocele major

**Figure:**
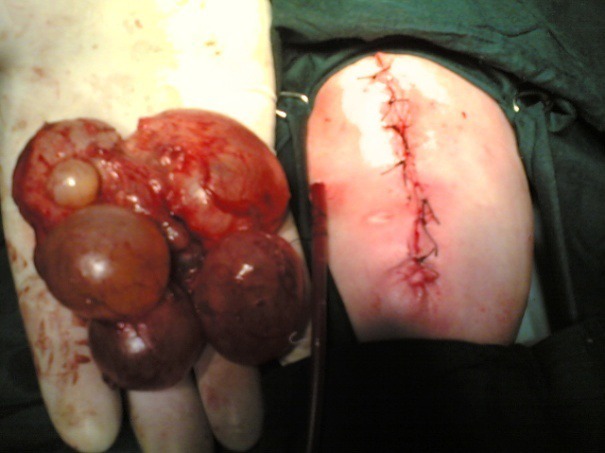
Figure 2: Right multicystic kidney and closure of omphalocele

**Case 2:**A 2-month-old male baby, previously operated on 2nd day of life for omphalocele minor, presented with abdominal distention associated with regurgitation of feed and irritability. On examination, there was a palpable mass which was soft, smooth, and slightly mobile. The erect abdominal radiograph showed a soft tissue shadow in the right iliac fossa pushing the bowel to the other side. CT scan of the abdomen showed huge cystic mass in the abdomen (Fig. 3). On exploration there was a cystic duplication of the terminal ileum which was excised along with resection of the bowel related to the cyst with end to end anastomosis. Patient is doing fine on follow-up.

**Figure:**
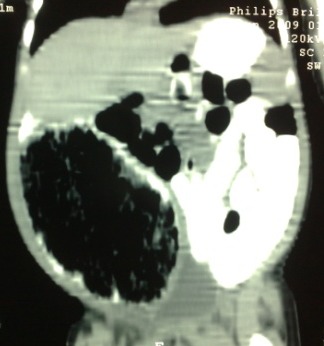
Figure 3: CT scan of the abdomen showing huge cyst in the right abdomen

**Figure:**
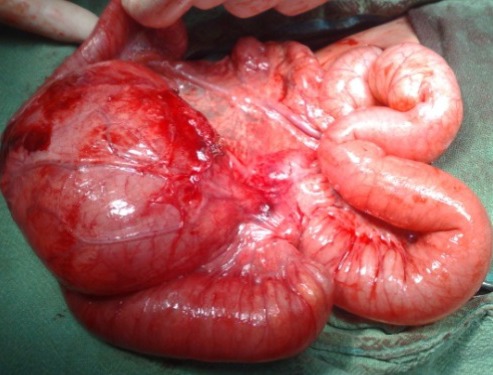
Figure 4: Communicating cystic duplication of the terminal ileum.

## DISCUSSION

Both of our cases add to the cases of omphalocele associated with intra-abdominal congenital anomalies. Only few cases of omphalocele have been associated with multicystic dysplastic kidney. Kawakita et al [2] reported a case of omphalocele associated with multiple anomalies including bilateral multicystic kidneys, atrial septal defects, and anomalies of the face and hands. The patient died of respiratory distress due to potter’s syndrome. Our case had unilateral renal anomaly and thus had good prognosis. Removal of dysplastic kidney further eased us in facial closure of the abdominal wall defect. Three cases of antenatally diagnosed enteric duplications have been reported in literature [3, 4]. In our 2nd case, the omphalocele was small and duplication cyst was missed owing to small defect. Later on, it presented with abdominal distension due to accumulation of bowel contents in the communicating duplication cyst of the terminal ileum. Other intra-abdominal anomalies are reported with omphalocele as malrotation, atresia, Meckels diverticulum, polycystic kidney and hydronephrosis.

## Footnotes

**Source of Support:** Nil

**Conflict of Interest:** None

